# Supplementary material for: Direction-Controlled Chemical Doping for Reversible G-Phonon Mixing in ABC Trilayer Graphene
Source: Sci Rep. 2015 Mar 9;5:8707. doi: 10.1038/srep08707 (PMC4352872; doi:10.1038/srep08707)
Supplement: Supplementary Information [file srep08707-s1.pdf]

Supplementary Information for

Direction-Controlled Chemical Doping for Reversible G-Phonon Mixing in ABC Trilayer Graphene

Kwanghee Park[1] and Sunmin Ryu[2]*
[1]Department of Applied Chemistry, Kyung Hee University, Yongin, Gyeonggi 446-701, Korea
[2]Department of Chemistry, Pohang University of Science and Technology (POSTECH), Pohang, Gyeongbuk 790-784, Korea

*E-mail: sunryu@postech.ac.kr

Contents

A. Spectral decomposition of 2D peaks of ABA and ABC trilayers

B. Raman maps of ABC-ABA 3L used for top-down charge doping

C. Doping-induced change in linewidths of G peaks

D. Top-down charge doping and its reversal in AB bilayers

E. Estimation of charge density and G peak intensity bifurcation

F. Supplementary references

A. Spectral decomposition of 2D peaks of ABA and ABC trilayers

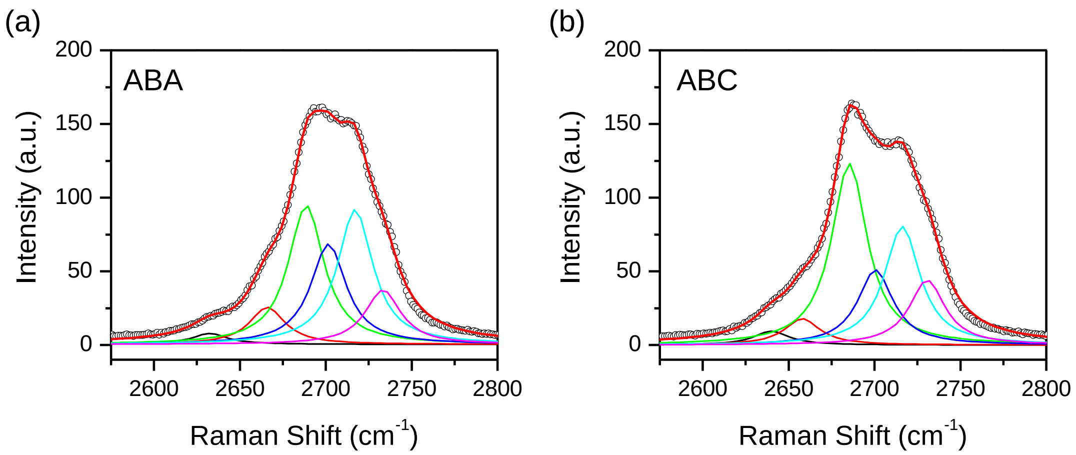

Figure S1. Spectral decomposition of 2D peaks of (a) ABA and (b) ABC trilayers with 6 Lorentzian functions with the linewidth fixed as 25 cm^{-1}. The excitation wavelength was 514 nm.

B. Raman maps of ABC-ABA 3L used for top-down charge doping

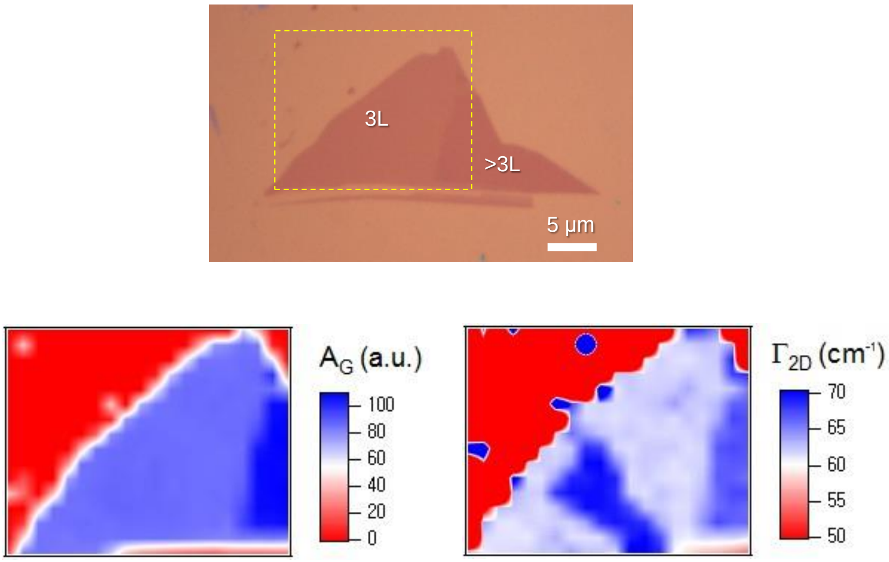

Figure S2. ABC-ABA 3L used for top-down charge doping. (a) Optical micrograph of 3L sample, where the region denoted '>3L' is thicker than 3L. (b) A_G-map of the pristine sample obtained from the dashed rectangle in (a). (c) Γ_{2D}-map obtained in its pristine state.

C. Doping-induced change in linewidths of G peaks

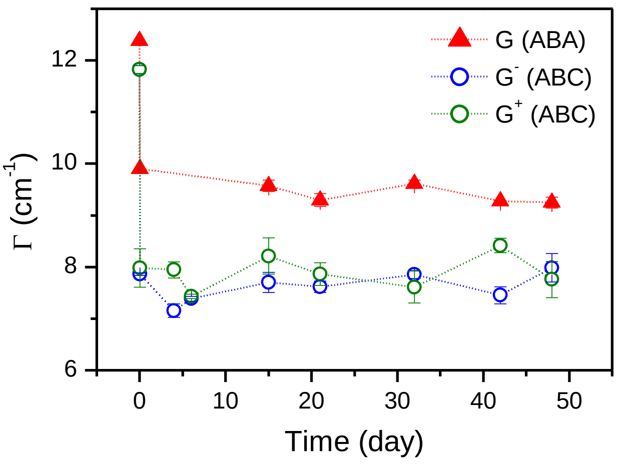

Figure S3. The linewidths of G peaks for ABA and ABC trilayers as a function of lapsed time since the exposure to I_2 vapor.

D. Top-down charge doping and its reversal in AB bilayers

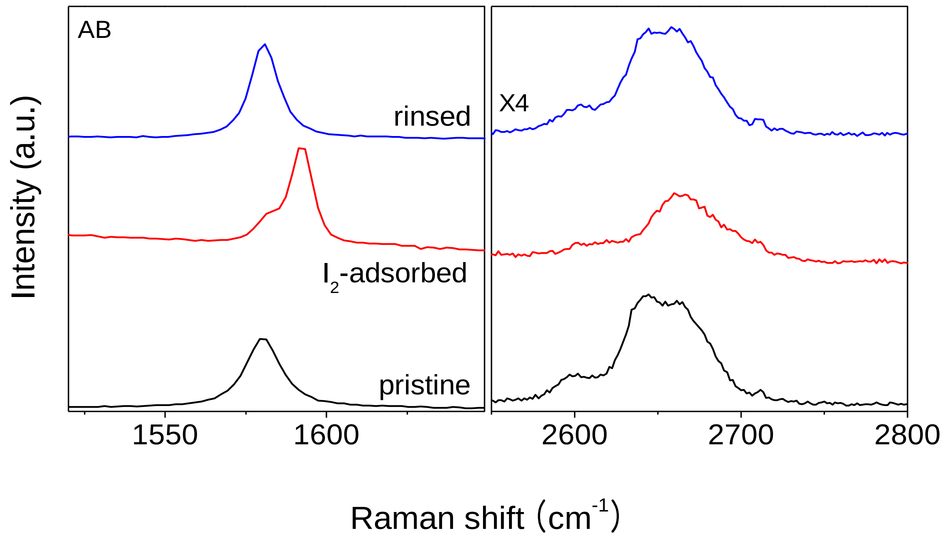

Figure S4. G and 2D Raman spectra of AB bilayers revealing the top-down charge doping and its reversal using I_2 as hole dopants. The excitation wavelength was 633 nm.

E. Estimation of charge density and G peak intensity bifurcation

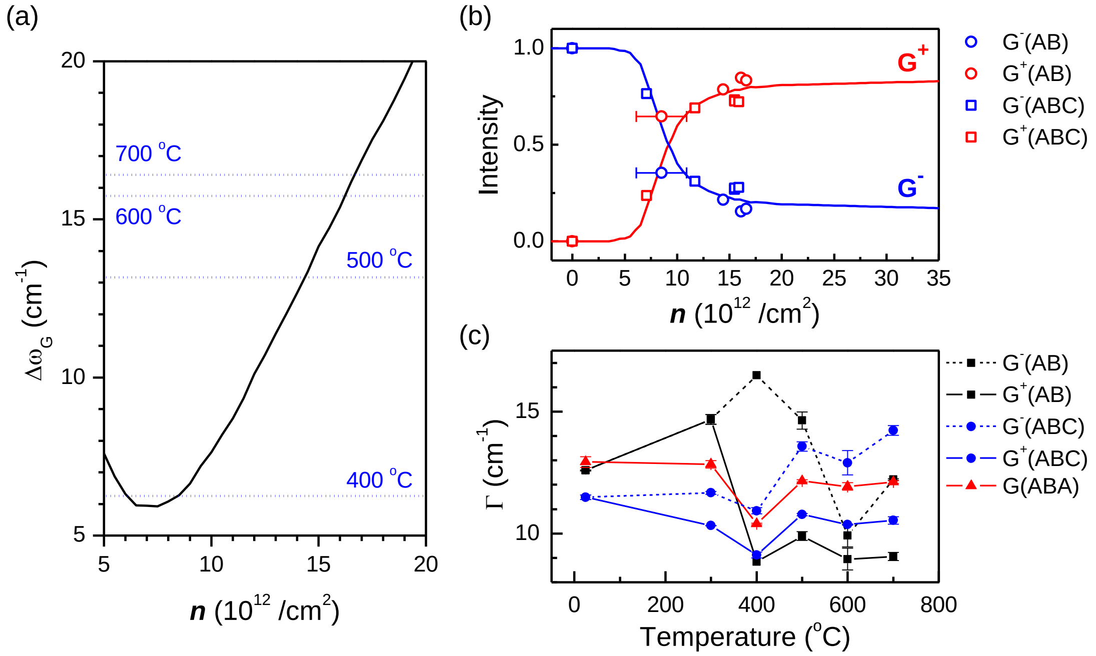

Figure S5. Estimation of charge density and G peak intensity bifurcation of the bottom-up-doped AB and ABC layers. (a) The G peak splitting ($\Delta\omega_G$) of AB layers as a function of hole density (***n***), where the solid and the dotted lines represent the theoretical calculation of Ando et al.[1] and the annealed AB layers, respectively. ***n*** of each sample can be determined from where the dotted line intersects the solid line. For the 400 °C case, the higher ***n*** of the two intersections was chosen since the intensity crossover from G$^-$ to G$^+$ has occurred (see Fig. S5b). The error bars in ***n*** for 400 °C case correspond to the difference between the two ***n*** values, representing the uncertainty associated with the current estimation. (b) The fractional G peak intensity of AB and ABC layers as a function of ***n***, where ***n*** of ABC layers were estimated assuming that $\Delta\omega_G$ of ABC layers obeys the same ***n***-dependence as that of AB layers. (c) The linewidth (Γ) of G peaks of AB, ABC and ABA layers as a function of the annealing temperature. Unlike the electrical gating experiment by Yan et al.,[2] the linewidth does not show the trend that the theory predicted.[1] The disagreement is attributed to the inhomogeneous broadening induced by the mechanical strain built during the annealing cycles.[3]

F. Supplementary references

1. Ando, T. & Koshino, M. Field effects on optical phonons in bilayer graphene. *J. Phys. Soc. Jpn.* **78**, 034709 (2009).
2. Yan, J., Villarson, T., Henriksen, E. A., Kim, P. & Pinczuk, A. Optical phonon mixing in bilayer graphene with a broken inversion symmetry. *Phys. Rev. B* **80**, 241417 (2009).
3. Lee, J. E., Ahn, G., Shim, J., Lee, Y. S. & Ryu, S. Optical separation of mechanical strain from charge doping in graphene. *Nat. Commun.* **3**, 1024 (2012).
